# Molecular Testing for Mycoplasma genitalium in the United States: Results from the AMES Prospective Multicenter Clinical Study

**DOI:** 10.1128/JCM.01125-19

**Published:** 2019-10-23

**Authors:** Charlotte A. Gaydos, Lisa E. Manhart, Stephanie N. Taylor, Rebecca A. Lillis, Edward W. Hook, Jeffrey D. Klausner, Carmelle V. Remillard, Melissa Love, Byron McKinney, Damon K. Getman

**Affiliations:** a Division of Infectious Diseases, Johns Hopkins University, Baltimore, Maryland, USA; b Departments of Epidemiology, Global Health, and Center for AIDS and STD, University of Washington, Seattle, Washington, USA; c Louisiana State University Health Sciences Center, New Orleans, Louisiana, USA; d Departments of Medicine, Epidemiology and Microbiology, University of Alabama, Birmingham, Birmingham, Alabama, USA; e UCLA Division of Infectious Diseases, Department of Medicine, David Geffen School of Medicine, University of California, Los Angeles, Los Angeles, California, USA; f Hologic, Inc., San Diego California, USA; Marquette University

**Keywords:** *Mycoplasma genitalium*, sexually transmitted infection, Aptima, Aptima *Mycoplasma genitalium* Evaluation Study (AMES)

## Abstract

A prospective multicenter clinical study involving subjects from 21 sites across the United States was conducted to validate the performance of a new *in vitro* diagnostic nucleic acid amplification test (NAAT) for the detection of Mycoplasma genitalium. Seven urogenital specimen types (*n* = 11,556) obtained from 1,778 females, aged 15 to 74 years, and 1,583 males, aged 16 to 82 years, were tested with the Aptima Mycoplasma genitalium assay, an investigational transcription-mediated amplification (TMA) NAAT for the detection of M. genitalium 16S rRNA. Infected status for enrolled subjects was established using results obtained from testing either self-collected vaginal swab or clinician-collected male urethral swab specimens with a composite reference method consisting of three transcription-mediated amplification NAATs targeting unique regions of M. genitalium 16S or 23S rRNA. M. genitalium prevalence was 10.2% in females and 10.6% in males; prevalence was high in both symptomatic and asymptomatic subjects for both sexes. Compared to the subject infected status standard, the investigational test had sensitivity and specificity estimates, respectively, of 98.9% and 98.5% for subject-collected vaginal swabs, 92.0% and 98.0% for clinician-collected vaginal swabs, 81.5% and 98.3% for endocervical swabs, 77.8% and 99.0% for female urine, and 98.2% and 99.6% for male urethral swabs, 88.4% and 97.8% for self-collected penile meatal swabs, and 90.9% and 99.4% for male urine specimens. For all seven specimen types, within-specimen positive and negative agreements between the investigational test and the composite reference standard ranged from 94.2% to 98.3% and from 98.5 to 99.9%, respectively. These results provide clinical efficacy evidence for the first FDA-cleared NAAT for M. genitalium detection in the United States.

## Abstract (precis)

A prospective multicenter clinical study involving subjects from 21 sites across the United States was conducted to validate the performance of a new *in vitro* diagnostic nucleic acid amplification test (NAAT) for the detection of Mycoplasma genitalium.

## INTRODUCTION

Mycoplasma genitalium is a fastidious bacterium in the class *Mollicutes*. Its minute 580-kb genome is the smallest known among prokaryotes capable of self-replication (1, 2). M. genitalium was first cultured in 1981 using urethral specimens from men with nongonococcal urethritis (NGU) (3). Slow growth *in vitro* and burdensome culture requirements have precluded routine diagnosis using this method (4). Nucleic amplification assay tests (NAATs) based on PCR and transcription-mediated amplification (TMA) chemistries have been necessary for the study of associations between infection in humans and disease (5–9). Since the availability of such molecular assays, the organism has been associated with many disease syndromes, such as urethritis in men and cervicitis and adverse reproductive sequelae such as endometritis and pelvic inflammatory disease in women. In addition to the cervix, vagina, and male urethra, M. genitalium is also found in the oropharynx and rectum (10–20). An association of M. genitalium infection with risk for HIV infection has been reported also (21). Increasing the concerns about treatment of the syndromes associated with M. genitalium are the reports of rising rates of resistance to azithromycin and moxifloxacin (22, 23), the primary agents used to treat these conditions. The overall importance of M. genitalium as a sexually transmitted pathogen has been comprehensively reviewed (24).

The development of the Aptima Mycoplasma genitalium (AMG) assay, an *in vitro* diagnostic (IVD) transcription-mediated amplification (TMA) assay that targets the 16S RNA of M. genitalium, has led to its experimental use to study the epidemiology and clinical outcomes associated with infection with the organism, as well as to a comparison with other molecular amplification assays (15, 16, 25–30). The assay has been used with sequencing to demonstrate high levels of macrolide antibiotic resistance in M. genitalium infections originating in the United States (31). Following receipt of the Conformité Européene (CE) mark from the European Union (32, 33), the AMG assay was clinically validated for detection of M. genitalium in urogenital specimens collected from subjects enrolled in a prospective, multicenter study encompassing multiple regions of the United States. The manuscript reports a summary of the U.S. study results.

## MATERIALS AND METHODS

### Study design and ethics approval.

This cross-sectional study was conducted in accordance with the ethical principles derived from the Declaration of Helsinki and Belmont Report and in compliance with the U.S. Food and Drug Administration (FDA) and Good Clinical Practice Guidelines (cGCP) set forth by the International Conference on Harmonization (ICH-E6). The study protocol (A10109-MGENPS-CSP-01) was approved by the local institutional review board at every site. Written informed consent was obtained from each subject at the time of enrollment, prior to specimen collection. Participants were compensated for study participation.

### Study population.

Sexually active female and male subjects of ≥14 years of age with (symptomatic) or without (asymptomatic) symptoms of sexually transmitted infections (STIs) (e.g., abnormal discharge, genital itching, pain/discomfort during sexual intercourse or urination, or pain/discomfort in groin or lower belly) were eligible for enrollment. Subjects were enrolled at 21 U.S. sites (clinical research centers and emergency medicine, family planning, public health, STI, family medicine/obstetric-gynecologic [OB-GYN] facilities) between July 2017 and April 2018. Exclusion criteria included antibiotic treatment (i.e., with macrolides, fluoroquinolones, tetracyclines, or clindamycin) within 21 days of enrollment or previous enrollment in this study.

### Sample collection.

Four specimens were collected in the clinic from each female subject in the following order: one self-collected first-catch urine specimen, one self-collected vaginal swab specimen, one clinician-collected vaginal swab specimen, and one clinician-collected endocervical swab specimen. Three specimens were collected in the clinic from each male subject in the following order: one self-collected penile meatal swab specimen, one clinician-collected urethral swab specimen, and one self-collected first-catch urine specimen. Vaginal and penile meatal specimens were collected using Aptima multitest swabs and placed in Aptima tubes containing specimen transfer medium (STM). Urethral and endocervical specimens were collected using an Aptima unisex swab and placed in Aptima tubes containing STM. First-catch urine specimens (i.e., approximately 20 to 30 ml of the initial urine stream collected in a urine collection cup free of any preservatives) were processed for testing using an Aptima urine specimen collection kit and placed in Aptima tubes containing urine transport medium (UTM).

### TMA and specimen testing.

The design, format, and comparative analytical performance of the AMG transcription-mediated amplification (TMA) assays and three alternate (Alt) TMA assays used for the composite reference standard have been described previously (34). All specimens were tested first with the AMG assay on an automated Panther system in one of three U.S. laboratories before being transported to Hologic for reference testing. Reference testing was performed using three research-use-validated alternate TMA assays developed by Hologic to capture, amplify, and detect unique regions of the 16S rRNA (Alt TMA assay-1) or 23S rRNA (Alt TMA assay-2 and Alt TMA assay-3) of M. genitalium; the Alt TMA assay-1 detected a different region of the 16S rRNA than the AMG assay. All three Alt TMA assays have similar analytical and clinical sensitivities (34). Alt TMA assays were performed on a Panther system (Alt TMA assays-1 and -2) or a manual direct tube sampling (DTS) system (Alt TMA-3) using validated laboratory-developed assay software. Each specimen was tested using Alt TMA assays -1 and -2; if the results of the two tests were discordant, the result from Alt TMA assay-3 testing was used as a tiebreaker. If two Alt TMA assay results were positive, the reference result was classified positive; if two Alt TMA results were negative, the reference result was negative (Table 1). Operators performing Alt TMA assays were blinded to AMG test results and all patient identifying information.

**TABLE 1:** Algorithm for establishing patient infected status using alternate TMA assay consensus results

Reference specimen result^*a*^			Patient infected status
Alt TMA assay-1^*b*^	Alt TMA assay-2	Alt TMA assay-3	
Positive	Positive	NA	Positive
	Negative	Invalid/missing	Unknown
	Negative	Positive	Positive
	Negative	Negative	Negative
	Invalid/missing	Invalid/missing	Unknown
	Invalid/missing	Positive	Positive
	Invalid/missing	Negative	Unknown
Negative	Positive	Invalid/missing	Unknown
	Positive	Positive	Positive
	Positive	Negative	Negative
	Negative	NA	Negative
	Invalid/missing	Invalid/missing	Unknown
	Invalid/missing	Positive	Unknown
	Invalid/missing	Negative	Negative
Invalid/missing	Positive	Invalid/missing	Unknown
	Positive	Positive	Positive
	Positive	Negative	Unknown
	Negative	Invalid/missing	Unknown
	Negative	Positive	Unknown
	Negative	Negative	Negative
	Invalid/missing	NA	Unknown

a The reference specimen is the urethral swab sample for male subjects and the self-collected vaginal swab sample for female subjects. NA, not applicable.

b Alt TMA, alternate transcription-mediated amplification.

### Statistical methods.

Prevalence (based on reference test result and patient infected status [PIS]), sensitivity, specificity, positive predictive value, and negative predictive value were calculated according to standard equations (35). Confidence intervals (CIs) for sensitivity and specificity were calculated using the score method. The confidence intervals for positive and negative predictive values were calculated using the exact method. Samples with inconclusive reference results and samples with invalid or missing investigational assay results were excluded from the analyses. The positive likelihood ratio (PLR) was calculated as sensitivity/(1 − specificity), and negative likelihood (NLR) was calculated as (1 − sensitivity)/specificity. Analyses were performed with SAS software (version 9.4; SAS Institute, Inc., Cary, NC).

### PIS.

To determine the clinical performance of the investigational assay in the assessed specimen types, Aptima TMA results were assessed relative to the patient infected status (PIS). The PIS was based on reference (composite Alt TMA assay) results from testing of the urethral swab sample for male subjects and of the patient-collected vaginal swab sample for female subjects; based on prior studies, vaginal swab and urethral samples are the most sensitive specimen types for the detection of M. genitalium (25, 36, 37). Table 1 shows the algorithm for establishing the PIS. Unless otherwise specified, for each specimen type, assay performance for the detection of M. genitalium was calculated relative to the PIS.

## RESULTS

### Study design and subject accountability.

**FIG 1:**
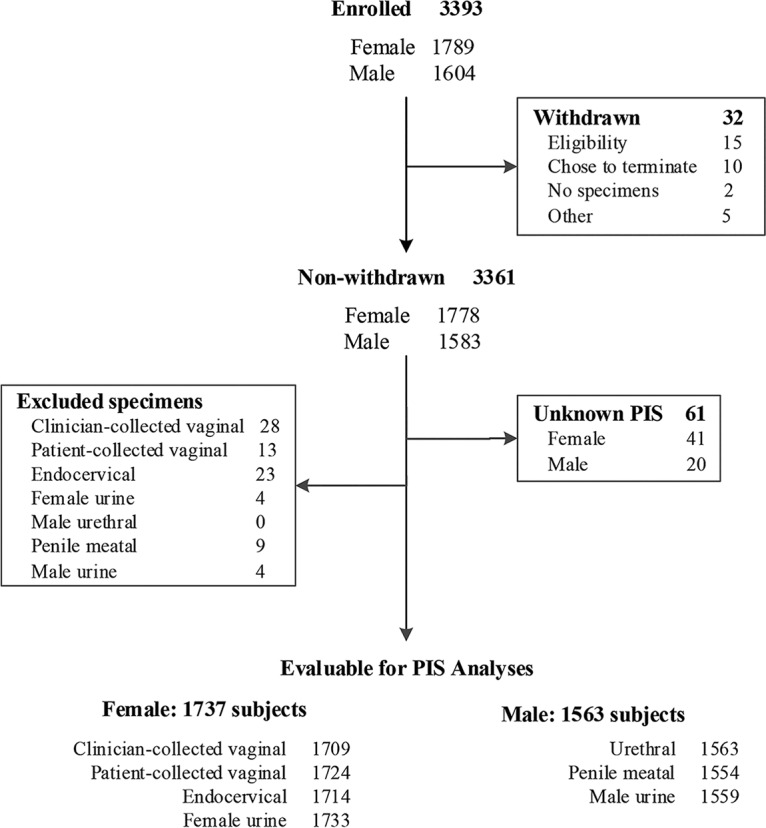
Overall study design and subject accountability. Subjects were not evaluable for the analysis versus PIS if they had an unknown PIS. Specimens with missing or invalid Aptima results were excluded from all analyses.

Figure 1 shows the patient accountability log, as well as the final numbers of evaluable samples for each specimen type. There were 3,393 subjects enrolled in the study, including 1,789 females and 1,604 males. Of these, 32 subjects were withdrawn for various reasons, including ineligibility or self-termination of participation. An additional 61 subjects with insufficient test results available for establishing a PIS were excluded, leaving 3,300 subjects (1,737 females and 1,563 males) evaluable for analysis. A total of 11,556 specimens were collected and analyzed using four female specimen types (*n* = 6,880) and three male specimen types (*n* = 4,676).

Demographic characteristics of enrolled subjects for sex, age, and race/ethnicity, as well as symptom status and M. genitalium prevalence for each group, are shown in Table 2. The majority of subjects were between 18 and 40 years of age (83.7% female; 70.1% male), Black (∼61% for both sexes), non-Hispanic (∼77% for both sexes), and from southeast or southwest clinical centers (∼74% for both sexes). For females, 60.6% were symptomatic; 55.4% of males were symptomatic. For females, M. genitalium prevalence was 11.6% and 7.9% among symptomatic and asymptomatic subjects, respectively; for males, M. genitalium prevalence was 12.0% and 8.8% among symptomatic and asymptomatic subjects, respectively. The highest prevalence of infection was in young adults of both sexes aged 18 to 20 years (female, 26.2%; male, 15.1%).

**TABLE 2:** Prevalence of M. genitalium urogenital infection by subject demographic status and geographic region

Category	No. of specimens	M. genitalium prevalence			
		Female		Male	
		No. positive/total no.	% positive (95% CI)	No. positive/total no.	% positive (95% CI)
Subject age (yr)					
15–17	4	0/3	0 (0.0–56.1)	0/1	0 (0.0–79.3)
18–20	242	39/149	26.2 (19.8–33.8)	14/93	15.1 (9.2–23.7)
21–30	1,379	94/805	11.7 (9.6–14.1)	81/574	14.1 (11.5–17.2)
31–40	929	36/500	7.2 (5.2–9.8)	53/429	12.4 (9.6–15.8)
41–50	345	6/157	3.8 (1.8–8.1)	11/188	5.9 (3.3–10.2)
51–60	298	1/94	1.1 (0.2–5.8)	5/204	2.5 (1.1–5.6)
61–70	90	0/25	0 (0.0–13.3)	1/65	1.5 (0.3–8.2)
71–82	13	0/4	0 (0.0–49.0)	0/9	0 (0.0–29.9)
All females (15–74)	1,737	176/1,737	10.1 (8.8–11.6)		
All males (16–82)	1,563			165/1,563	10.6 (9.1–12.2)
Symptom status^*a*^					
Symptomatic	1,919	122/1,053	11.6 (9.8–13.7)	104/866	12.0 (10.0–14.3)
Asymptomatic	1,381	54/684	7.9 (6.1–10.2)	61/697	8.8 (6.9–11.1)
Subject race/ethnicity^*b*^					
Asian	47	5/29	17.2 (7.6–34.5)	0/18	0 (0.0–17.6)
Black	2,025	127/1,059	12.0 (10.2–14.1)	125/966	12.9 (11.0–15.2)
White	1,131	40/591	6.8 (5.0–9.1)	37/540	6.9 (5.0–9.3)
Unknown/other race	146	6/79	7.6 (3.5–15.6)	6/67	9.0 (4.2–18.2)
Hispanic	720	23/381	6.0 (4.1–8.9)	23/339	6.8 (4.6–10.0)
Non-Hispanic	2,556	151/1,347	11.2 (9.6–13.0)	140/1,209	11.6 (9.9–13.5)
Unknown ethnicity	24	2/9	22.2 (6.3–54.7)	2/15	13.3 (3.7–37.9)
U.S. geographic area^*c*^					
Mid-Atlantic	260	16/142	11.3 (7.1–17.5)	13/118	11.0 (6.6–17.9)
Midwest	288	23/190	12.1 (8.2–17.5)	14/98	14.3 (8.7–22.6)
Northeast	225	13/106	12.3 (7.3–19.9)	11/119	9.2 (5.2–15.8)
Northwest	65	0/12	0 (0.0–24.2)	3/53	5.7 (1.9–15.4)
Southeast	1,424	72/703	10.2 (8.2–12.7)	84/721	11.7 (9.5–14.2)
Southwest	1,038	52/584	8.9 (6.9–11.5)	40/454	8.8 (6.5–11.8)

a Symptom status was determined based on subject-reported symptoms.

b Subjects could report multiple responses.

c Mid-Atlantic: Maryland, North Carolina, and Washington DC; Midwest: Indiana, Michigan, Nebraska, and Ohio (2 sites); Northeast: Connecticut and New Jersey; Northwest: Washington; Southeast: Alabama, Georgia, Florida (3 sites), and Louisiana; Southwest: California (2 sites) and Texas (2 sites).

### Prevalence and clinical performance.

The M. genitalium prevalence and the clinical performance of the investigational assay for the detection of M. genitalium are shown in Table 3 for each specimen type overall and by symptom status. In females, prevalence was lowest (7.9%) in endocervical and urine samples from asymptomatic subjects and highest (11.6%) in urine samples and patient-collected vaginal specimens from symptomatic subjects. In males, prevalence was lowest in urine specimens (8.7%) from asymptomatic subjects and highest (12%) in urethral swab and urine specimens from symptomatic subjects. The overall prevalence of M. genitalium was similar for clinician-collected vaginal swabs, patient-collected vaginal swab, endocervical swab, and female urine specimens (10.2, 10.2, 10.1, and 10.2%, respectively) and for urethral swab, penile-meatal swab, and male urine specimens (10.6, 10.6, and 10.5%, respectively).

**TABLE 3:** Clinical performance characteristics of the Aptima Mycoplasma genitalium assay in urogenital specimens from female and male subjects

Specimen type and subject symptom status^*a*^	No. of specimens	Prevalence (%)	Sensitivity (% [95% CI])	Specificity (% 95% CI)	PPV (95% CI)^*b*^	NPV (95% CI)^*c*^	PLR (95% CI)^*d*^	NLR (95% CI)^*e*^
Clinician-collected vaginal swab								
Sym	1,040	11.5	93.3 (87.4–96.6)	97.6 (96.4–98.4)	83.6 (77.3–88.8)	99.1 (98.3–99.6)	39.0 (26.2–60.8)	0.07 (0.03–0.13)
Asym	669	8.1	88.9 (77.8–94.8)	98.7 (97.5–99.3)	85.7 (75.8–92.9)	99.0 (98.0–99.6)	68.3 (35.6–148.8)	0.11 (0.04–0.23)
Overall	1,709	10.2	92.0 (86.9–95.1)	98.0 (97.2–98.6)	84.2 (79.1–88.6)	99.1 (98.5–99.5)	47.0 (33.4–68.8)	0.08 (0.05–0.13)
Patient-collected vaginal swab								
Sym	1,047	11.6	100 (96.9–100)	98.1 (96.9–98.8)	87.1 (81.1–91.9)	100 (99.6–100)	51.4 (32.7–86.5)	0.00 (0.00–0.03)
Asym	677	8.0	96.3 (87.5–99.0)	99.0 (97.9–99.6)	89.7 (80.4–95.7)	99.7 (98.9–100)	100 (47.4–258.2)	0.04 (0.00–0.13)
Overall	1,724	10.2	98.9 (95.9–99.7)	98.5 (97.7–99.0)	87.8 (83.1–91.7)	99.9 (99.5–100)	63.8 (43.4–97.9)	0.01 (0.00–0.04)
Endocervical swab								
Sym	1,046	11.5	84.2 (76.6–89.6)	98.2 (97.1–98.9)	85.6 (79.1–90.8)	98.0 (97.0–98.7)	45.9 (29.1–76.4)	0.16 (0.1–0.24)
Asym	669	7.9	75.5 (62.4–85.1)	98.5 (97.2–99.2)	81.6 (70.3–90.2)	97.9 (96.8–98.8)	51.7 (27.5–107.2)	0.25 (0.14–0.39)
Overall	1,715	10.1	81.5 (75.1–86.6)	98.3 (97.5–98.8)	84.4 (78.9–89.1)	97.9 (97.2–98.5)	48.3 (33.3–72.7)	0.19 (0.13–0.25)
Female urine								
Sym	1,051	11.6	79.5 (71.5–85.7)	98.4 (97.4–99.0)	86.6 (80.0–91.8)	97.3 (96.3–98.2)	49.2 (30.4–85.7)	0.21 (0.14–0.29)
Asym	682	7.9	74.1 (61.1–83.9)	99.8 (99.1–100)	97.6 (88.7–99.9)	97.8 (96.7–98.7)	465.2 (91.6–15,195.2)	0.26 (0.15–0.4)
Overall	1,733	10.2	77.8 (71.1–83.3)	99.0 (98.3–99.4)	89.5 (84.3–93.6)	97.5 (96.8–98.2)	75.8 (47.5–128.6)	0.22 (0.17–0.29)
Male urethral swab								
Sym	866	12.0	98.1 (93.3–99.5)	99.9 (99.3–100)	99.0 (94.9–100)	99.7 (99.1–100)	747.4 (136.8–27,947.9)	0.02 (0.00–0.07)
Asym	697	8.8	98.4 (91.3–99.7)	99.2 (98.2–99.7)	92.3 (84.0–97.3)	99.8 (99.2–100)	125.1 (54.7–369.0)	0.02 (0.00–0.09)
Overall	1,563	10.6	98.2 (94.8–99.4)	99.6 (99.1–99.8)	96.4 (92.7–98.6)	99.8 (99.4–100)	228.8 (106.8–605.2)	0.02 (0.00–0.05)
Penile meatal swab								
Sym	865	11.9	89.3 (81.9–93.9)	97.8 (96.5–98.6)	84.4 (77.5–90.0)	98.5 (97.6–99.2)	40.0 (25.4–66.7)	0.11 (0.06–0.19)
Asym	689	8.9	86.9 (76.2–93.2)	97.9 (96.5–98.8)	80.3 (70.8–88.1)	98.7 (97.7–99.4)	42.0 (25.0–75.9)	0.13 (0.06–0.24)
Overall	1,554	10.6	88.4 (82.6–92.5)	97.8 (96.9–98.5)	82.9 (77.4–87.6)	98.6 (97.9–99.1)	40.9 (29.0–59.8)	0.12 (0.07–0.18)
Male urine								
Sym	866	12.0	89.4 (82.0–94.0)	99.1 (98.1–99.6)	93.0 (86.9–96.9)	98.6 (97.6–99.3)	97.3 (48.5–228.4)	0.11 (0.06–0.18)
Asym	693	8.7	93.3 (84.1–97.4)	99.7 (98.9–99.9)	96.6 (89.0–99.5)	99.4 (98.5–99.8)	295.4 (85.6–2,229.8)	0.07 (0.02–0.16)
Overall	1,559	10.5	90.9 (85.5–94.4)	99.4 (98.8–99.7)	94.3 (90.0–97.2)	98.9 (98.3–99.4)	140.8 (76.2–294.7)	0.09 (0.05–0.15)

a Symptom status was determined based on subject-reported symptoms. Asym, asymptomatic; Sym, symptomatic.

b PPV, positive predictive value.

c NPV, negative predictive value.

d PLR, positive likelihood ratio.

e NLR, negative likelihood ratio.

Overall sensitivity of the investigational test for detection of M. genitalium-infected subjects was ≥90% for clinician- and patient-collected vaginal and male urethral swab specimens and male urine specimens, 88.4% for penile-meatal swab specimens, 81.5% for endocervical specimens, and 77.8% for female urine specimens. Overall specificity was ≥97.8% for all specimen types. The combination of investigational and reference assay M. genitalium results for all subjects with a conclusive PIS status and valid AMG assay results are shown in Tables S1 and S2 in the supplemental material for female and male urogenital specimens, respectively. Sensitivity and specificity estimates were similar in asymptomatic and symptomatic subjects for each specimen type. Assay performance was similar among races and ethnicities for each specimen type (Tables S3 and S4).

In the absence of results from FDA-approved assays for M. genitalium detection for performance comparison, positive (PLR) and negative (NLR) likelihood ratios were also calculated. By symptom status, estimates of the PLR in female specimen types ranged from 39.0 (95% CI, 26.2 to 60.8) for clinician-collected vaginal swab specimens from symptomatic subjects to 465.2 (95% CI, 91.6 to 15,195.2) for female urine specimens from asymptomatic subjects. For male specimens, PLR estimates ranged from 40.0 (95% CI, 25.4 to 66.7) for penile meatal swab specimens from symptomatic subjects to 747.4 (95% CI, 136.8 to 27,947.9) for urethral swab specimens from symptomatic subjects. For all specimen types, NLR ratios were less than 0.26. Together, these results demonstrated highly relevant and statistically significant increases in knowledge based on positive and negative AMG assay results in all specimen types.

To investigate the effect of the clinical specimen matrix on investigational assay performance for specimens other than the male urethral swab and self-collected vaginal swab, a comparison of AMG assay and Alt TMA assay results within the same specimen type was performed (Table 4). Infected specimen status was determined for these analyses using the same general rules as for the PIS, except that the infected status was determined based on the composite Alt TMA reference result for that specimen instead of in comparison to the patient-collected vaginal swab specimen (for women) or the urethral swab specimen (for men). Positive percent agreement (PPA) using the specimen infected status standard was >95% for all specimen types except female urine, for which the PPA was 94.6% in symptomatic subjects and 93.2% in asymptomatic subjects. Negative percent agreement (NPA) was >98% in all specimen types.

**TABLE 4:** Specimen-specific agreement of the Aptima Mycoplasma genitalium assay in urogenital specimens from female and male subjects

Specimen type and subject symptom status^*a*^	No. of specimens	Comparison of assay results (no.)				Positive % agreement (95% CI)	Negative % agreement (95% CI)
		Aptima positive		Aptima negative			
		Reference positive	Reference negative	Reference negative	Reference positive		
Clinician-collected vaginal swab							
Sym	1,050	123	12	913	2	98.4 (94.4–99.6)	98.7 (97.7–99.3)
Asym	679	52	5	621	1	98.1 (90.1–99.7)	99.2 (98.1–99.7)
Overall	1,729	175	17	1,534	3	98.3 (95.2–99.4)	98.9 (98.3–99.3)
Patient-collected vaginal swab							
Sym	1,047	121	18	908	0	100 (96.9–100)	98.1 (96.9–98.8)
Asym	677	52	6	617	2	96.3 (87.5–99.0)	99.0 (97.9–99.6)
Overall	1,724	173	24	1,525	2	98.9 (95.9–99.7)	98.5 (97.7–99.0)
Endocervical swab							
Sym	1,057	115	4	935	3	97.5 (92.8–99.1)	99.6 (98.9–99.8)
Asym	677	48	3	624	2	96.0 (86.5–98.9)	99.5 (98.6–99.8)
Overall	1,734	163	7	1,559	5	97.0 (93.2–98.7)	99.6 (99.1–99.8)
Female urine							
Sym	1,074	106	7	955	6	94.6 (88.8–97.5)	99.3 (98.5–99.6)
Asym	700	41	2	654	3	93.2 (81.8–97.7)	99.7 (98.9–99.9)
Overall	1,774	147	9	1,609	9	94.2 (89.4–96.9)	99.4 (98.9–99.7)
Male urethral swab							
Sym	866	102	1	761	2	98.1 (93.3–99.5)	99.9 (99.3–100)
Asym	697	60	5	631	1	98.4 (91.3–99.7)	99.2 (98.2–99.7)
Overall	1,563	162	6	1,392	3	98.2 (94.8–99.4)	99.6 (99.1–99.8)
Penile meatal swab							
Sym	870	101	8	756	5	95.3 (89.4–98.0)	99.0 (97.9–99.5)
Asym	693	61	6	623	3	95.3 (87.1–98.4)	99.0 (97.9–99.6)
Overall	1,563	162	14	1,379	8	95.3 (91.0–97.6)	99.0 (98.3–99.4)
Male urine							
Sym	874	99	2	770	3	97.1 (91.7–99.0)	99.7 (99.1–99.9)
Asym	704	60	0	643	1	98.4 (91.3–99.7)	100 (99.4–100)
Overall	1,578	159	2	1,413	4	97.5 (93.9–99.0)	99.9 (99.5–100)

a Symptom status was determined based on subject-reported symptoms. Asym, asymptomatic; Sym, symptomatic.

**FIG 2:**
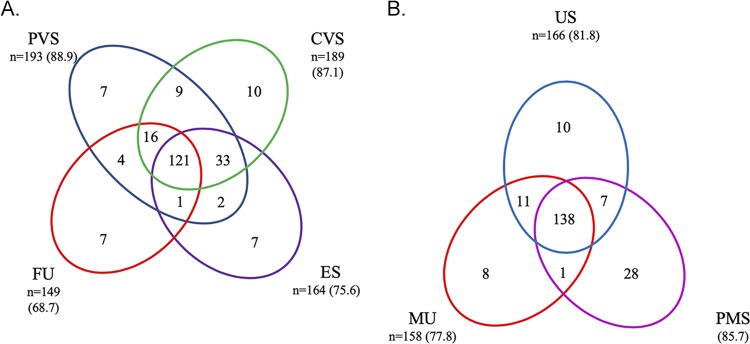
Joint distribution of Aptima Mycoplasma genitalium assay positive results in clinical specimens from female (*n* = 217) (A) and male (*n* = 203) (B) subjects. Specimen category values are the number (percent) of test-positive subjects for each specimen type. CVS, clinician-collected vaginal swab; ES, endocervical swab; FU, female urine; MU, male urine; PMS, penile meatal swab; PVS, patient-collected vaginal swab; US, male urethral swab.

Figure 2 depicts the distribution of female and male urogenital specimens with unique and shared positive AMG assay results. The majority of subjects were positive for M. genitalium in more than one specimen type (85.7% for female specimens; 77.3% for male specimens). However, for both female and male subjects, a minority of subjects had positive AMG assay results in only one sample type (e.g., 7/193 patient-collected vaginal swab specimens; 28/174 penile meatal swab specimens). Most subjects had positive AMG results for two or more specimen types; 121 (55.8%) females and 138 (68%) males reported positive AMG results in all specimen types assessed.

**FIG 3:**
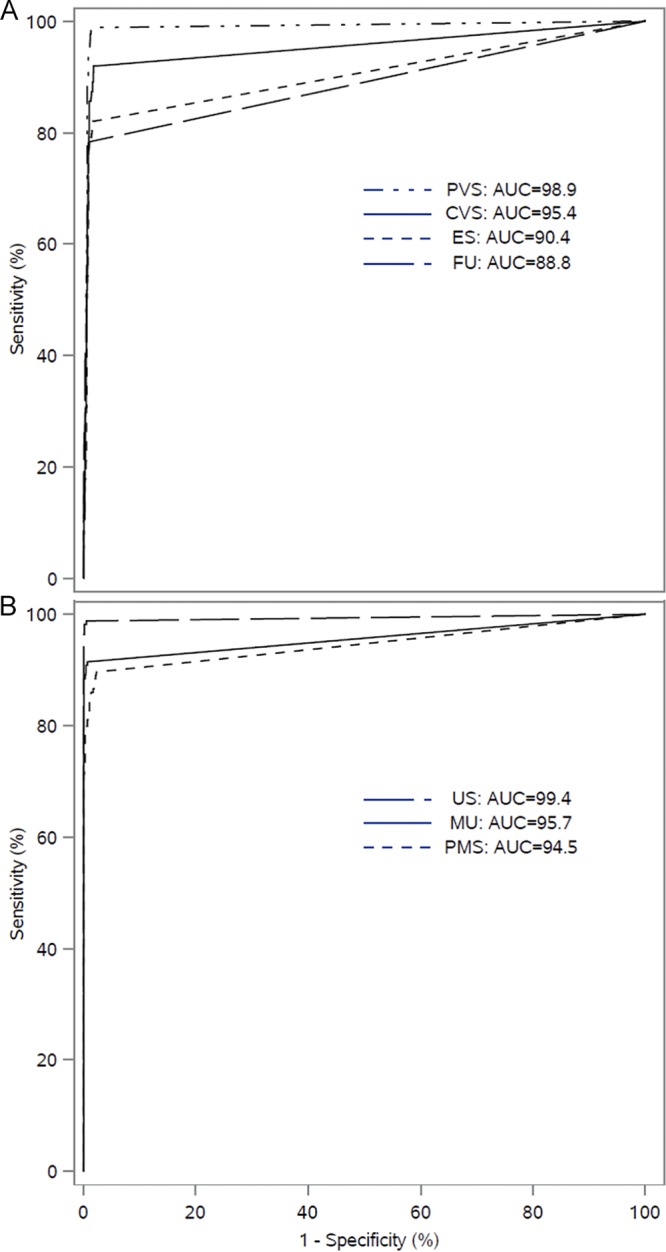
ROC curve analysis of female (A) and male (B) clinical specimen types for detection of M. genitalium-infected subjects. CVS, clinician-collected vaginal swab; ES, endocervical swab; FU, female urine; MU, male urine; PMS, penile meatal swab; PVS, patient-collected vaginal swab; US, male urethral swab.

Receiver operating characteristic (ROC) curve analysis was performed on the female and male urogenital specimens (Fig. 3). The area under the curve (AUC) estimates ranged from 88.8 for female urine specimens to 98.9 for patient-collected vaginal swab specimens and from 94.5 for male penile meatal swab specimens to 99.4 for male urethral swab specimens.

## DISCUSSION

This study reports the results of a prospective multicenter clinical performance evaluation of the Aptima Mycoplasma genitalium assay for detection of M. genitalium. These results provide clinical efficacy evidence for the first IVD NAAT for M. genitalium detection in the United States. Following completion of this study, the assay received FDA clearance (510k# DEN180047) for the detection of M. genitalium in patient- and clinician-collected urogenital specimens from symptomatic and asymptomatic subjects, including minors. The FDA clearance will now allow laboratories to test for M. genitalium in a wide variety of urogenital specimens without having to develop and validate laboratory-developed tests (30) and will allow clinicians to easily detect this pathogen in their patients.

The results of this study are in concert with previous research study results that have highlighted the accuracy of this assay (25, 29–32). The prevalence of M. genitalium observed in this study in symptomatic and asymptomatic persons is consistent with that previously reported for the various types of specimens used for detection in males and females (9, 15–17, 26, 31). The performance of the assay in multiple specimen types should allow clinicians to choose the specimen type most appropriate for individual patient management. For females, the vaginal swab had the best performance, with self-collected vaginal swabs having higher sensitivity (98.9%) than clinician-collection vaginal swabs (92%). Female urine and endocervical swabs showed somewhat lower sensitivity (81.5% and 77.8%, respectively), consistent with previous reports (25, 36); however, as with the vaginal swab specimens, these two specimen types had positive and negative likelihood ratios significantly different from unity, indicating high probabilities for diagnostic accuracy using these two specimen types. In males, the urethral swab demonstrated high sensitivity of 98.2%, and detection rates of infections using male urine and the self-collected penile-meatal swab were similar, giving clinicians options for sampling male patients. From the ROC analysis, all female and male specimen types had AUC values greater than 88%.

To assess the effect of anatomic site-specific infection on sensitivity and specificity estimates determined using the PIS standard, a comparison of AMG assay and Alt TMA assay results within the same specimen type was performed. Positive percent agreement with the specimen-infected status was >95% for all specimen types except female urine, for which the PPA was 94.6% in symptomatic subjects and 93.2% in asymptomatic subjects. Negative percent agreement was >98% for all specimen types. This suggests the somewhat lower diagnostic value (PLR and NLR) and/or diagnostic accuracy (sensitivity and specificity) estimates associated with some specimen types such as female urine may be due to anatomic site-specific infections (e.g., urinary tract-positive/genital tract-negative) rather than specimen matrix effects on assay performance.

While there is little debate that M. genitalium is sexually transmitted, there is wide discrepancy in the prevalence detected in the general population compared to the prevalence in patients seeking care from many types of clinics. In lower-risk populations, an M. genitalium prevalence of approximately 1% to 3% has been reported in both men and women (38, 39). In higher-risk populations attending STI clinics, prevalences of 9% to 24% in men and 11% to 16% in women have been reported (13–16). The debate is whether asymptomatic infection with M. genitalium is associated with disease and the future development of adverse sequelae. In this AMES study, the prevalences in symptomatic males and females were 12% and 11.6%, respectively, whereas, in asymptomatic persons the prevalences were 8.8% and 7.9%, respectively, demonstrating a prevalence not very different from the prevalence of chlamydia in symptomatic women seen in many health care settings. There are many observational reports that disease manifestations of persistent urethritis (25), cervicitis (16, 17, 40–42), and even pelvic inflammatory disease (PID) and other adverse reproductive sequelae (17, 43, 44) are associated with M. genitalium detection in males and females. An unmet need for understanding the public health significance of infection with M. genitalium includes prospective trials that demonstrate that screening and treating asymptomatic persons prevents adverse reproductive sequelae in women and persistent urethritis and sequelae in asymptomatic men although such studies are likely to be costly.

A major concern with the increasing use of diagnostic testing and treatment of infected patients is the high antibiotic resistance of M. genitalium to azithromycin, the first-line antibiotic used to treat urogenital infections (22, 23, 31, 33). Macrolide resistance rates of greater than 40% are common worldwide and appear to be increasing; this is especially important for the treatment of PID caused by M. genitalium, where standard syndromic treatment may fail (45–48). It will be important to incorporate antibiotic resistance detection for macrolides and other antibiotic classes into future screening algorithms for M. genitalium as part of the larger antibiotic stewardship efforts needed for the clinical management of all STIs.

Our study has limitations. We did not collect oropharyngeal or rectal specimens, potentially important sources of M. genitalium infection and transmission, and we do not have coinfection data for other STIs. Additionally, there is no information about the antibiotic resistance status of M. genitalium-positive subjects, which is an important clinical consideration (31, 46–48). We also lack extensive history on the sexual orientation of subjects and their sex partners, HIV status, or other risk factors. However, this study was not designed to address those questions.

Strengths of this study include adherence to cGCP regulations to enroll a large, diverse cohort from six geographic areas of the United States, including 15 states and the District of Columbia and representing patients attending multiple clinical practice types. In addition, multiple sample types were collected from each patient, providing clinicians options for patient management. Finally, the composite reference standard used in this study consisted of a consensus result from three validated TMA assays targeting M. genitalium rRNAs, eliminating potential bias due to differences in sensitivities between the investigational test and reference assays (34).

In summary, we now have an FDA-cleared IVD assay that can be used to detect M. genitalium urogenital infections in men and women using cervical, vaginal, urethral, penile-meatal, and urine specimens. Future research will be required to further define the pathogenicity of M. genitalium, the best treatment algorithms, and its significance when detected in asymptomatic persons.

## Supplementary Material

Supplemental file 1
