# Supplementary material for: Molecular Testing for Mycoplasma genitalium in the United States: Results from the AMES Prospective Multicenter Clinical Study
Source: J Clin Microbiol. 2019 Oct 23;57(11):e01125-19. doi: 10.1128/JCM.01125-19 (PMC6813011; doi:10.1128/JCM.01125-19)
Supplement: Supplemental file 1 [file JCM.01125-19-s0001.pdf]

Supplemental Table S1. ***Mycoplasma genitalium*** **infected status for female urogenital specimens.**

	Self-collected Vaginal Swab			Aptima Mycoplasma genitalium Assay				Symptom Status[b]	
**Patient Infected Status**	**Alt TMA Assay -1**	**Alt TMA Assay -2**	**Alt TMA Assay -3[a]**	**Self-collected Vaginal Swab**	**Clinician-collected Vaginal Swab**	**Endocer-vical Swab**	**Urine**	**Sym**	**Asym**
Infected	+	+	N/A	+	+	+	+	71	25
Infected	+	+	N/A	+	+	+	-	14	8
Infected	+	+	N/A	+	+	-	+	7	8
Infected	+	+	N/A	+	+	-	-	4	0
Infected	+	+	N/A	+	-	+	-	0	1
Infected	+	+	N/A	+	-	-	+	1	0
Infected	+	+	N/A	+	-	-	-	0	1
Infected	+	+	N/A	+	-	NR	+	1	0
Infected	+	+	N/A	+	NR	+	+	1	0
Infected	+	+	N/A	-	+	-	-	0	1
Infected	+	+	N/A	NR	NR	+	+	1	0
Infected	+	-	+	+	+	+	+	0	1
Infected	+	NR	+	+	+	+	+	1	2
Infected	-	+	+	+	+	+	+	10	2
Infected	-	+	+	+	+	+	-	2	0
Infected	-	+	+	+	+	-	+	1	0
Infected	-	+	+	+	+	-	-	1	0
Infected	-	+	+	+	+	NR	-	1	0
Infected	-	+	+	+	-	+	+	1	0
Infected	-	+	+	+	-	-	+	2	1
Infected	-	+	+	+	-	-	-	3	1
Infected	-	+	+	+	-	NR	-	0	1
Infected	-	+	+	-	-	-	-	0	1
Infected	NR	+	+	+	+	+	+	0	1

	Self-collected Vaginal Swab			Aptima Mycoplasma genitalium Assay				Symptom Status[b]	
Patient Infected Status	Alt TMA Assay -1	Alt TMA Assay -2	Alt TMA Assay -3[a]	Self-collected Vaginal Swab	Clinician-collected Vaginal Swab	Endocer-vical Swab	Urine	Sym	Asym
Not infected	+	-	-	+	+	+	+	1	0
Not infected	+	-	-	+	+	+	-	2	0
Not infected	-	+	-	+	+	+	+	3	0
Not infected	-	+	-	+	+	+	-	1	2
Not infected	-	+	-	+	+	-	-	0	1
Not infected	-	+	-	+	-	-	-	1	0
Not infected	-	+	-	-	+	-	-	1	1
Not infected	-	+	-	-	-	-	+	1	0
Not infected	-	-	-	-	-	-	-	2	0
Not infected	-	-	N/A	+	+	+	+	4	0
Not infected	-	-	N/A	+	+	+	-	3	1
Not infected	-	-	N/A	+	+	-	-	1	2
Not infected	-	-	N/A	+	-	+	-	1	0
Not infected	-	-	N/A	+	-	-	-	1	0
Not infected	-	-	N/A	-	+	-	-	6	1
Not infected	-	-	N/A	-	-	+	-	2	5
Not infected	-	-	N/A	-	-	-	+	4	1
Not infected	-	-	N/A	-	-	-	-	845	568
Not infected	-	-	N/A	-	-	-	NR	2	2
Not infected	-	-	N/A	-	-	NR	-	5	9
Not infected	-	-	N/A	-	NR	-	+	1	0
Not infected	-	-	N/A	-	NR	-	-	9	11
Not infected	-	-	N/A	-	NR	NR	-	0	3
Not infected	-	-	N/A	NR	-	+	-	0	1

	Self-collected Vaginal Swab			Aptima Mycoplasma genitalium Assay				Symptom Status[b]	
**Patient Infected Status**	**Alt TMA Assay -1**	**Alt TMA Assay -2**	**Alt TMA Assay -3[a]**	**Self-collected Vaginal Swab**	**Clinician-collected Vaginal Swab**	**Endocer-vical Swab**	**Urine**	**Sym**	**Asym**
Not infected	-	-	N/A	NR	-	-	-	5	4
Not infected	-	-	N/A	NR	NR	NR	-	0	1
Not infected	-	NR	-	-	-	-	-	6	5
Not infected	NR	-	-	-	-	-	+	1	0
Not infected	NR	-	-	-	-	-	-	22	10
Not infected	NR	-	-	-	-	NR	-	0	1
Not infected	NR	-	-	-	NR	-	-	1	0
Not infected	NR	-	-	NR	-	-	-	0	1

Asym = asymptomatic; N/A = not applicable; NR = no result, Sym = symptomatic

[a] Alt TMA #3 results are not applicable if results of Alt TMA assays #1 and #2 are in agreement; some samples may have been tested unnecessarily with Alt TMA assay #3. [b]Symptom status is determined based on subject-reported symptoms.

Supplemental Table S2. ***Mycoplasma genitalium*** **infected status for male urogenital specimens.**

	Urethral Swab			Aptima Mycoplasma genitalium Assay			Symptom Status[b]	
**Patient Infected Status**	**Alt TMA Assay #1**	**Alt TMA Assay #2**	**Alt TMA Assay #3[a]**	**Urethral Swab**	**Penile Meatal Swab**	**Urine**	**Sym**	**Asym**
Infected	+	+	+	+	+	+	1	0
Infected	+	+	N/A	+	+	+	83	49
Infected	+	+	N/A	+	+	-	4	0
Infected	+	+	N/A	+	+	NR	0	1
Infected	+	+	N/A	+	-	+	7	3
Infected	+	+	N/A	+	-	-	3	1
Infected	+	+	N/A	+	NR	-	1	0
Infected	+	+	N/A	-	-	-	1	0
Infected	+	NR	+	+	+	+	1	1
Infected	-	+	+	+	+	-	1	0
Infected	-	+	+	+	-	-	0	1
Infected	-	+	+	-	+	-	1	0
Infected	-	+	+	-	-	-	0	1
Infected	NR	+	+	+	+	+	1	2
Infected	NR	+	+	+	-	+	0	1
Infected	NR	+	+	+	-	-	0	1
Not infected	-	+	-	+	+	-	0	1
Not infected	-	+	-	+	-	-	0	2
Not infected	-	+	-	-	+	-	1	0
Not infected	-	+	-	-	-	-	2	3
Not infected	-	-	-	-	-	-	1	0
Not infected	-	-	N/A	+	+	-	1	0
Not infected	-	-	N/A	+	-	-	0	2
Not infected	-	-	N/A	-	+	+	1	0

	Urethral Swab			Aptima Mycoplasma genitalium Assay			Symptom Status[b]	
**Patient Infected Status**	**Alt TMA Assay #1**	**Alt TMA Assay #2**	**Alt TMA Assay #3[a]**	**Urethral Swab**	**Penile Meatal Swab**	**Urine**	**Sym**	**Asym**
Not infected	-	-	N/A	-	+	-	14	11
Not infected	-	-	N/A	-	-	+	6	2
Not infected	-	-	N/A	-	-	-	721	589
Not infected	-	-	N/A	-	-	NR	0	3
Not infected	-	-	N/A	-	NR	-	0	8
Not infected	-	NR	-	-	+	-	0	1
Not infected	-	NR	-	-	-	-	7	5
Not infected	NR	-	-	-	-	-	8	9

Asym = asymptomatic; N/A = not applicable; NR = no result; Sym = symptomatic

[a] Alt TMA #3 results are not applicable if results of Alt TMA assays #1 and #2 are in agreement; some samples may have been tested unnecessarily with Alt TMA assay #3. [b]Symptom status is determined based on subject-reported symptoms.

Supplemental Table S3. Sensitivity and specificity of the Aptima Mycoplasma genitalium assay using female specimen types, by race and ethnicity.

	Percent (n/N) [95% CI]							
	PVS		CVS		ES		FU	
Category	Sens	Spec	Sens	Spec	Sens	Spec	Sens	Spec
Asian	100.0<br>(5/5)<br>[56.6-100.0]	100.0<br>(23/23)<br>[85.7-100.0]	100.0<br>(5/5)<br>[56.6-100.0]	100.0<br>(24/24)<br>[86.2-100.0]	100.0<br>(5/5)<br>[56.6-100.0]	100.0<br>(24/24)<br>[86.2-100.0]	80.0<br>(4/5)<br>[37.6-96.4]	100.0<br>(24/24)<br>[86.2-100.0]
Black	100.0<br>(126/126)<br>[97.0-100.0]	97.8<br>(903/923)<br>[96.7-98.6]	92.8<br>(116/125)<br>[86.9-96.2]	97.6<br>(893/915)<br>[96.4-98.4]	80.8<br>(101/125)<br>[73.0-86.7]	97.7<br>(901/922)<br>[96.5-98.5]	77.2<br>(98/127)<br>[69.1-83.6]	98.7<br>(917/929)<br>[97.8-99.3]
White	95.0<br>(38/40)<br>[83.5-98.6]	99.5<br>(546/549)<br>[98.4-99.8]	87.5<br>(35/40)<br>[73.9-94.5]	98.7<br>(535/542)<br>[97.4-99.4]	79.5<br>(31/39)<br>[64.5-89.2]	99.3<br>(538/542)<br>[98.1-99.7]	80.0<br>(32/40)<br>[65.2-89.5]	99.3<br>(546/550)<br>[98.1-99.7]
Unknown/Other	100.0<br>(6/6)<br>[61.0-100.0]	98.6<br>(72/73)<br>[92.6-99.8]	100.0<br>(6/6)<br>[61.0-100.0]	98.6<br>(72/73)<br>[92.6-99.8]	83.3<br>(5/6)<br>[43.6-97.0]	97.3<br>(71/73)<br>[90.5-99.2]	83.3<br>(5/6)<br>[43.6-97.0]	100.0<br>(73/73)<br>[95.0-100.0]
Hispanic	95.7<br>(22/23)<br>[79.0-99.2]	99.4<br>(354/356)<br>[98.0-99.8]	87.0<br>(20/23)<br>[67.9-95.5]	98.3<br>(348/354)<br>[96.4-99.2]	72.7<br>(16/22)<br>[51.8-86.8]	99.2<br>(353/356)<br>[97.6-99.7]	69.6<br>(16/23)<br>[49.1-84.4]	99.2<br>(354/357)<br>[97.6-99.7]
Non-Hispanic	99.3<br>(149/150)<br>[96.3-99.9]	98.1<br>(1164/1186)<br>[97.2-98.8]	92.6<br>(138/149)<br>[87.3-95.8]	98.0<br>(1151/1175)<br>[97.0-98.6]	82.6<br>(123/149)<br>[75.7-87.8]	98.0<br>(1156/1179)<br>[97.1-98.7]	78.8<br>(119/151)<br>[71.6-84.6]	98.9<br>(1180/1193)<br>[98.1-99.4]

CVS = Clinician-collected vaginal swab; ES = endocervical swab; FU = female urine; PVS = patient-collected vaginal swab

Supplemental Table S4. Sensitivity and specificity of the Aptima Mycoplasma genitalium assay using male specimen types, by race and ethnicity.

Category	Percent (n/N) [95% CI]					
	US		PMS		MU	
	Sens	Spec	Sens	Spec	Sens	Spec
Race/Ethnicity						
Asian	NC (0/0)	100.0 (18/18) [82.4-100.0]	NC (0/0)	100.0 (18/18) [82.4-100.0]	NC (0/0)	100.0 (18/18) [82.4-100.0]
Black	99.2 (124/125) [95.6-99.9]	99.4 (836/841) [98.6-99.7]	90.3 (112/124) [83.8-94.4]	97.7 (817/836) [96.5-98.5]	90.3 (112/124) [83.8-94.4]	99.0 (830/838) [98.1-99.5]
White	94.6 (35/37) [82.3-98.5]	99.6 (501/503) [98.6-99.9]	81.1 (30/37) [65.8-90.5]	98.4 (492/500) [96.9-99.2]	91.9 (34/37) [78.7-97.2]	99.8 (502/503) [98.9-100.0]
Unknown/Other	100.0 (6/6) [61.0-100.0]	98.4 (60/61) [91.3-99.7]	100.0 (6/6) [61.0-100.0]	91.8 (56/61) [82.2-96.4]	100.0 (6/6) [61.0-100.0]	100.0 (61/61) [94.1-100.0]
Hispanic	95.7 (22/23) [79.0-99.2]	100.0 (316/316) [98.8-100.0]	65.2 (15/23) [44.9-81.2]	98.1 (308/314) [95.9-99.1]	86.4 (19/22) [66.7-95.3]	99.7 (314/315) [98.2-99.9]
Non-Hispanic	98.6 (138/140) [94.9-99.6]	99.4 (1063/1069) [98.8-99.7]	92.1 (128/139) [86.4-95.5]	97.7 (1039/1063) [96.7-98.5]	91.4 (128/140) [85.6-95.0]	99.3 (1059/1067) [98.5-99.6]

MU = male urine; PMS = penile meatal swab; US = urethral swab
